# Prolonged labour associated with lower expression of syndecan 3 and connexin 43 in human uterine tissue

**DOI:** 10.1186/1477-7827-4-24

**Published:** 2006-05-04

**Authors:** Ann Hjelm Cluff, Birgitta Byström, Aurelija Klimaviciute, Camilla Dahlqvist, Gvido Cebers, Anders Malmström, Gunvor Ekman-Ordeberg

**Affiliations:** 1 Department of Woman and Child Health, Division for Obstetrics and Gynaecology, Karolinska University Hospital Solna, S-171 76 Stockholm, Sweden; 2 Department of Clinical Neuroscience, Division of Drug Dependent Research, Karolinska Institute, S-171 76 Stockholm, Sweden; 3 Department of Experimental Medical Science, C13 BMC, Lund University, S-221 84 Lund, Sweden

## Abstract

**Background:**

Prolonged labour is associated with greater morbidity and mortality for mother and child. Connexin 43 is a major myometrial gap junction protein found in human myometrium. Syndecan 3 seems to prevail in the human uterus among heparan sulphate proteoglycans, showing the most significant increase during labour. The aims of the present study were to investigate syndecan 3 and connexin 43 mRNA expressions and protein distributions in human uterine tissue during normal and prolonged labour.

**Methods:**

Uterine isthmic biopsies were collected from non-pregnant (n = 7), term pregnant women not in labour (n = 14), in normal labour (n = 7) and in prolonged labour (n = 7). mRNA levels of syndecan 3 and connexin 43 were determined by real time RT-PCR. The localization and expression were demonstrated by immunohistochemistry and confocal microscopy.

**Results:**

In women with prolonged labour, the mRNA expressions of syndecan 3 and Connexin 43 were considerably lower than the expression level at normal labour (p < 0.05). In term-pregnant tissue, the expression of syndecan 3 and connexin 43 did not differ significantly compared to non-pregnant and normal labour. The immunoreactivity of syndecan 3 was strong at normal labour, in contrast to prolonged labour, where both a weaker expression and an irregular distribution were detected. The immunoreactivity of connexin 43 increased until term and further stronger staining occurred at normal labour. At prolonged labour, the immunoreactivity was weaker and more unevenly distributed. At labour, a co-localization of syndecan 3 and connexin 43 could be demonstrated in the smooth muscle by confocal microscopy.

**Conclusion:**

The high expression of syndecan 3 and connexin 43 and their co-localization to the smooth muscle bundles during normal labour, together with the significant reduction in prolonged labour, may indicate a role for these proteins in the co-ordination of myometrial contractility.

## Background

The knowledge of the mechanisms controlling myometrial contractility has increased over the past decades, [1,2]. Understanding the complex process of labour is important as emergency caesarean sections following prolonged labour have an increased rate of maternal and foetal morbidity and mortality [3].

Normal labour is defined as uterine contractions that occur with a frequency, intensity and duration sufficient to cause dilation and effacement of the cervix [4]. To attain the synchronized contractions required for normal labour, the uterine muscle is dependent on a functioning cell-to-cell communication acquired by the action potential propagation [5]. The formation of gap junctions is essential, since these provide the necessary electrical coupling and the passage of ions and small molecules between neighbouring cells [6] Intercellular communication via gap junctions is of broad biological significance in many tissues, and several studies have shown how gap junctions increase in number and size in the myometrium prior to labour [7,8]. The mechanisms regulating the synthesis and assembly of gap junctions and their function are known to be related to many proteins [9].

Connexin 43 is a major myometrial gap junction protein found in human myometrium [6,10]. Known factors that influence gap junction formation and function are mechanical load, estrogens, nitric oxide, intracellular Ca^2+ ^levels and proteoglycans [11-17]. Several studies of other tissues also suggests that the extracellular matrix (ECM) that surrounds the smooth muscle cells plays an important role in the regulation of the connexin expression [18-20]. Furthermore, there is some evidence in vitro for an ECM-induced gap junction communication [21].

The ECM in the uterus undergoes pronounced remodelling during pregnancy and labour. In the corpus, the collagen concentration decreases significantly until term pregnancy [22]. In earlier investigations, we have registered higher uterine and cervical concentrations of collagen in women with protracted labour compared to those in normal labour [23]. The proteoglycan composition also undergoes changes during pregnancy and labour. We have previously demonstrated that decorin, the most abundant proteoglycan in the human uterus, decreases to 60% at term compared to the non – pregnant tissue [24]. An increase in the concentration of heparan sulphate proteoglycan (HSPG) occurs during labour, while the concentration of small proteoglycans remains constant [24]. The following HSPGs have been identified: syndecan 2, 3, 4, glypican 1 and perlecan. All are closely associated with the smooth muscle bundles. Among the HSPGs, syndecan 3 is dominant in the human uterus, showing the most significant increase during labour [25].

The aims of the present study were to investigate syndecan 3 and connexin 43 mRNA expressions and protein distributions in human uterine tissue during normal and prolonged labour.

## Materials and methods

### Subjects

The patients were allocated in four groups. The non-pregnant group consisted of seven women with a mean age of 43.4 years (range 40–48) and a mean parity of 2.1 (range 1–4). They were all menstruating and had no medication. All underwent hysterectomy due to benign indications.

In the term pregnant group fourteen women with a mean age of 33.0 years (range 21–44) and a mean parity of 0.4 (range 0–1) were included. They were delivered by elective caesarean section before the onset of labour at a mean gestational age of 38+5 days (range 38+3 to 39+0). The indications were breech presentation, pelvic disproportion or humanitarian.

The third group consisted of seven women with a mean age of 33.5 years (range 24 – 41), a mean parity of 0.2 (range 0–2) and a mean gestational age of 39+3 (range 36–41+5). They all had a spontaneous labour with normal progress without oxytocin augmentation. Their mean cervical dilation was 5.2 cm (range 4–7 cm). Caesarean sections were performed due to foetal indications.

The final group had seven nulliparous women with a mean age of 30.3 years (range 21 – 36) and a mean gestational age of 39+3 weeks (38+2–41+4). All but one was nulliparous. They were subjected to a caesarean section due to primary and/or secondary arrest of labour. Their mean cervical dilation was 5.5 cm (range 3 – 10 cm) and all received intravenous infusion of oxytocin for more than 3 hours.

The biopsies of about 300–400 mg were collected from the lower uterine segment of the human uterus. Every biopsy was divided in two. One was immediately frozen on dry ice and then stored at -70°C. The other part of the biopsy was for immunohistochemical analyses. The Local Ethics Committee at the Karolinska Hospital approved the study and all women gave their informed consent to participate in the study.

### Materials

RNA-later^® ^– ICE was obtained from Ambion Inc. (Huntingdon, Cambridgeshire UK). Trizol^® ^reagent was obtained from Invitrogen (Carlsbad, CA, USA). Random hexamer primers pd (N)_6 _was purchased from Amersham Pharmacia, Bioscience corp. (Uppsala, Sweden). Superscript RNAse H was purchased from Invitrogen, Life Technologies (Carlsbad, CA, USA).

A LightCycler Instrument real time PCR system (Roche, Basel, Switzerland) was used for the RT-PCR analysis, using LightCycler-DNA Master SYBR Green I (Roche, Basel, Switzerland).

A polyclonal antibody against Connexin 43 (71–0700 and 35–5000) and rabbit anti Syndecan-3 Mid (36–2400) were supplied by Zymed Laboratories Inc. (San Francisco, USA). A monoclonal antibody, anti human in mouse against Syndecan 3 was a kind gift from Professor Guido David, Leuwen, Belgium [26-28]. Avidin-biotin detection system Vectastein ABC-elite was purchased from Vector Lab. (Burlingame, CA. USA). All other chemicals were of analytical grade.

Alexa Fluor 633 goat anti-rabbit IgG and Alexa Fluor 488 rabbit anti-mouse antibodies were purchased from Molecular Probes (Eugene, OR, USA)

### RNA extraction and cDNA preparation

Total RNA was extracted from the frozen biopsies (thawed in RNA-later^® ^– ICE), using a Trizol reagent kit. The procedure was carried out according to the manufacturer's instructions. The quantification of total RNA was performed by measuring absorbance at OD_260 _(Eppendorf BioPhotometer, Hamburg, Germany).

The quality of total RNA was controlled by running 1.5% agarose-gel in TBE-buffer, and assessed as acceptable if strong and intact bands of 28S RNA and 18S rRNA were identified with ethidium bromide.

1 μg total RNA from each sample was reverse-transcripted using 200 ng random hexamer primer pd (N)_6 _in PCR-grade water (total 12 μl) incubated for 5 min in 65°C. The sample was then chilled on ice. 7 μl mixture of 5× buffer 0,1 mM DTT and 40 units RNAsin was added and incubated at 42°C for 2 min. 200 U of Superscript™ II RNAse H^- ^Reverse Transcriptase was added and incubated for 10 min. in room temperature, 50 min in 42°C and finally 10 min in 70°C. All samples were chilled immediately and stored in -70°C until use.

### Real time RT-PCR

Real-Time PCR was performed employing Roche Light Cycler Instrument.

30 μl PCR grade water was added to all samples before further standard cDNA and sample cDNA dilutions.

For the RT-PCR, 2 μl each of the standard cDNA dilutions (1:10, 1:100 and 1:1000) or the study samples were added to individual Light Cycler glass capillaries. DNTPs, Hot-Start Taq polymerase, reaction buffer, and SYBR Green I dye were supplied in the Light Cycler DNA Master SYBR Green I kit, of which 2 μl per capillary was added. The reaction was supplemented with 5 mM Mg^2+ ^and a 0.5 μM (final concentration) of each gene-specific primer. Reaction mixtures were brought to 20 μl with sterile water. Gene-specific primers for Syndecan 3 and for Connexin 43 were made at Invitrogen Life Technologies, Sweden. Sequences for the gene-specific primers are presented in Table 1.

**Table 1:** Sequences, annealing temperature and size for the gene-specific primers

Genes	Primers	Annealing temp.	Size bp
Syndecan 3	5'-ACC CCA ACT CCA GAG ACC TT-3' 5'-TTA CTC CAC CAT CGA CAC CC-3'	60°C	159 bp
Connexin 43 (Cx 43)	5'-GGC GTT AAG GAT CGG GTT AA-3' 5'-CGA CGA CCT GTA CTT AAT GTC GGT-3'	60°C	363 bp
28S	5'-TGCAGATCTTGGTGGTAGTAGC-3' 3'-AGAGCCAATCCTTATCCCGAAGTT-5'	58°C	552 bp

PCR was performed for 45 cycles (15 s of denaturising at 95°C; 10 s of annealing at 60°C; and 20 s of extension at 72°C). Syndecan 3 and Connexin 43 data were normalized to the mRNA levels of housekeeping gene 28S from the same cDNA preparation. The mRNA expressions are presented as the quotients Syndecan 3/28S and Connexin 43/28S respectively.

Aliquots of the reaction products were run on 1.5% agarose gel containing ethidium bromide (0.5 μM/ml) in order to mark and visualize the PCR products and verify their size.

### Immunohistochemistry

The uterine biopsies were immediately fixed for two hours at 4°C in a solution of 14% saturated picric acid and 4% formalin. The biopsies were rinsed for at least 24 hours in Sorensen's buffer, containing 10% sucrose, 0.01% NaN_3 _(sodium azide) and 0.02% Bacitracin. The biopsies were then frozen in to -20°C. Serial cryostat sections of 14 nm were air dried and stored in to -20°C. The endogenous peroxidase activity was eliminated by pre-treatment with 0.3% hydrogen peroxide in methanol for 30 minutes followed by washing in PBS/BSA (0.05%). Blocking was also performed with normal sera, horse and goat respectively, for 30 minutes. After that the sections were incubated, without prior rinsing, with the primary antibodies, Syndecan 3 and Connexin 43, over night in 4°C. After rinsing with PBS/BSA the slides were incubated with the secondary antibodies, horse anti mouse and goat anti rabbit respectively, for 30 minutes. Then, after rinsing, detection of the antibody was performed with the Avidin Biotin Complex system for 30 minutes and the brown positive staining was developed with DAB (Diaminobenzedine-kit from Vector). The blue counterstaining was made with Haematoxylin. The antibody concentrations were for syndecan 1.25 μg/ml (1:800) and for Connexin 43 2.5 μg/ml (1:200). The immunoreactivity was estimated and graded blindly into four groups: none (-), weak (+), intermediate (++) or strong (+++) staining by three independent observers (AHC + GEO + BS). The magnification was 1 × 200 and 1 × 400 with oil.

### Confocal microscopy

Sections from uterine biopsies prepared as above were thawed and washed 3 times in 20 mM Tris and 150 mM NaCl, pH 7.5 containing 0.05% Tween-20 for 5 min. Blocking was carried out in the same buffer supplemented with 3% BSA for 45 min. The wash step was repeated once. Sections were then incubated with rabbit anti-Syndecan-3 Mid diluted 1:100 with 10 mM Tris pH 7.3 containing 2% bovine serum albumin and 0.02% Na-azide. After 5 times 5 minutes of washing with 20 mM Tris HCl and 150 mM NaCl, pH 7.5 containing 0.05% Tween-20, the sections were incubated with an Alexa Fluor 633 goat anti-rabbit IgG antibody diluted 1:400 with blocking buffer for 1 hour. After washing as above, the sections were then incubated with a mouse anti-Connexin 43 using a dilution of 1:200 for 1 hour. The sections were then washed as above and treated with 1: 1000 diluted Alexa Fluor 488 rabbit anti-mouse IgG antibody diluted in blocking buffer for 1 hour. Finally, after washing, the sections were air dried and mounted with 2 drops of Fluorescent Mounting Medium and a cover glass. The sections were examined using a LeicaTCS PII Confocal System. Using a 40× or 63× objective, the sections were corrected for autofluorescence and "over bleeding" and then sectioned into 0.52 μm thin optical sections.

### Statistical method

The Mann-Whitney test was used to evaluate the differences between the groups, as the data was not normally distributed. A p-value < 0.05 was considered statistically significant. Calculations were performed employing STATISTICA 6.0 software (StatSoft Inc, Tulsa, OK, USA).

## Results

**Figure 1:**
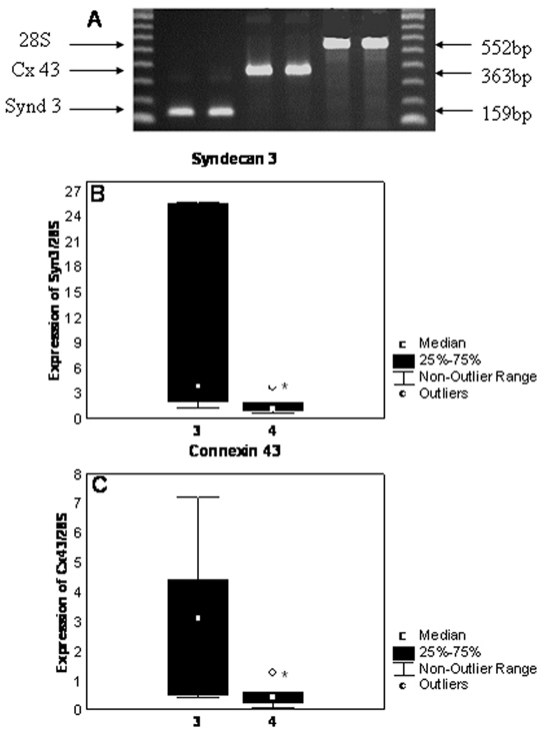
**Box and whisker plots representing mRNA expression of Syndecan 3 and Connexin 43 in normal labour (gr. 3) and in prolonged labour (gr. 4)**. A. Estimation of RT-PCR product size by 1.5% agarose gel electrophoresis. Molecular weight standards contain 10 bands ranging from 100 to 1000 bp. All amplicons had expected size. mRNA expression of (B) Syndecan 3 and (C)Connexin 43. The levels of mRNA expression were normalized to housekeeping gene 28S expression. * – statistically significant differences (p < 0.05) between the group compared to normal labour.

In women with prolonged labour the mRNA expressions of syndecan 3 and Connexin 43 were significantly lower than the expression level at normal labour (p < 0.05) (Fig. 1B, C). In term-pregnant tissue, the expression of syndecan 3 and connexin 43 did not differ significantly compared to non-pregnant and normal labour, but there was a tendency of highest expression in normal labour (data not shown).

Separation of the amplicons after real time PCR revealed that all amplicons did have the expected size (Fig. 1A).

**Figure 2:**
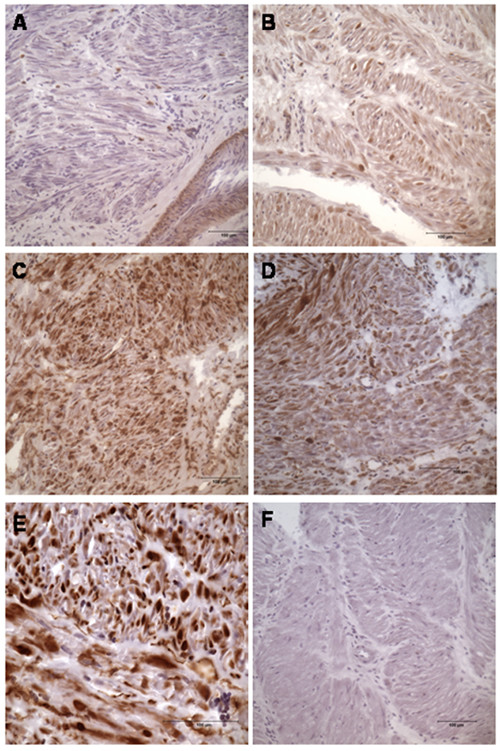
**Immunohistochemical staining of Syndecan 3 in uterine tissue at different stages of pregnancy**. (A) Non-pregnant (group 1), (B) term-pregnant (group 2), (C) women in normal labour (group 3), (D) in prolonged labour (group 4), (E) in normal labour, magnification 1 × 400 with oil, (F) negative control. Magnification in A, B, C, D and F is 1 × 200. Positive staining is brown. The grading of staining is presented in Table 2.

Immunohistochemistry showed that the majority of syndecan 3 was localized in the smooth muscle bundles (Fig. 2), a finding that agrees with results in previous studies [25]. In non-pregnant tissue the expression was scarce, but an obvious increase of syndecan 3 was found in term pregnant and an even larger increase at normal labour. Here, a clear and very strong staining was seen in individual muscle fibres (Fig. 2, Table 2). In the group with prolonged labour, the expression in the smooth muscle tissue was also fairly strong. However, an interesting pattern was seen within the muscle in this group, where some bundles were strongly positive whereas others only had a very weak staining of syndecan 3 (Fig. 2, Table 2).

**Table 2:** The grading of syndecan 3 staining

Syndecan 3	Gr.1	Gr.2	Gr.3	Gr.4
Smooth muscle	+	++	+++	1–3 (varies)
ECM	+	+	++	+
Vessels	+	+	+	+

Grading: weak (+), intermediate (++) and strong (+++). Groups studied: non-pregnant (Gr. 1), term pregnant (Gr. 2), in normal labour (Gr. 3), in prolonged labour (Gr. 4).

**Figure 3:**
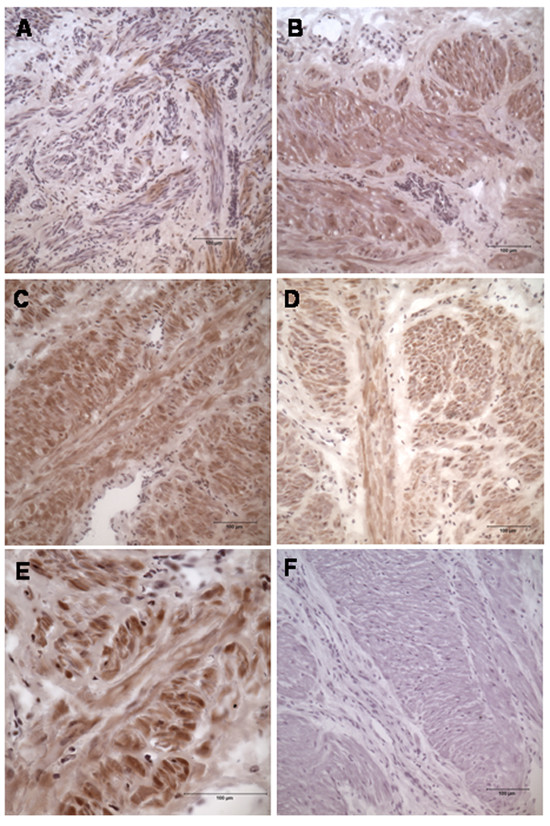
**Immunohistochemical staining of Connexin 43 at different stages of pregnancy**. (A) Non-pregnant (group 1), (B) term-pregnant (group 2), (C) in normal labour (group 3), D in prolonged labour (group 4), (E) in normal labour, magnification 1 × 400 with oil, (F) negative control. Magnification in A, B, C, D and F is 1 × 200 Positive staining is brown. The grading of staining is presented in Table 3.

Connexin 43 did stain the non-pregnant tissue but very weakly, especially in the muscle bundles (Fig. 3, Table 3). The term-pregnant tissue did show a stronger reactivity, reaching a maximal expression in the muscle bundle obtained at normal labour. Overall, the staining was much more even than that of syndecan 3. In the group with prolonged labour, the immunoreactivity of connexin 43 was also sparser compared to normal labour (Fig. 3, Table 3).

**Table 3:** The grading of connexin 43 staining

Connexin 43	Gr.1	Gr.2	Gr.3	Gr.4
Smooth muscle	+	++	+++	++
ECM	+	+	+	+
Vessels	+	+	+	+

Grading: weak (+), intermediate (++) and strong (+++). Groups studied: non-pregnant (Gr. 1), term pregnant (Gr. 2), in normal labour (Gr. 3), in prolonged labour (Gr. 4).

**Figure 4:**
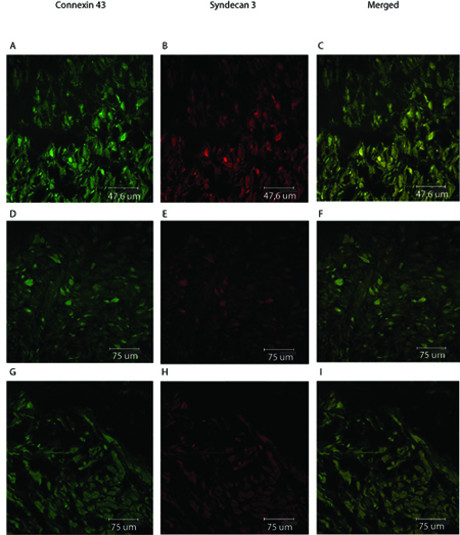
**Co-localization of Connexin 43 and Syndecan 3 in uterine tissue during normal labour**. Sections from uterine tissue from women in normal labour (panel A-C), patients at term (panel D-F) and patients with prolonged labour (panel G-I) were obtained and prepared as described above. A monoclonal antibody against syndecan 3 was added, followed by Alexa Fluor 633 goat anti-rabbit IgG antibody. After that, a Connexin 43 antibody followed by Alexa Fluor 488 rabbit anti-mouse IgG antibody was added. The excitation for Connexin 43 (panel A, D and G) and Syndecan 3 (panel B, E and H) are shown. The merged picture demonstrates the co-localization in the tissue (panel C, F and I).

The localizations of Syndecan 3 and Connexin 43 were studied by confocal microscopy, in order to investigate the basis of functional effects between them. Three patients in normal labour were examined. The connexin 43 distribution was mainly confined to the muscle bundles (Fig. 4 A). Changing the plane of focus yielded the observation that a significant part of the staining was found on the cell surface. The syndecan 3 distribution was similar to that of connexin 43, with a distribution to the muscle fibres that was more pronounced on some of the fibres (Fig. 4 B). A merging of the excitation pattern gave a very clear co-localization image of patients in normal labour, where the yellow staining was found on the surface of the smooth muscle fibres (Fig. 4 C). Focusing the instrument at various depths of the specimens further supported this conclusion. A considerably smaller co-localization was noted in sections from term patients and patients with prolonged labour (Fig 4 D–I).

## Discussion

Our results from the distribution studies of syndecan 3 and Connexin 43 showed a clear co-variation with the highest immunoreactivity at normal labour. The expression in tissue obtained from patients with protracted labour showed a weaker and, as far as Syndecan 3 is concerned, an uneven and patchy distribution. The irregular distribution of Syndecan 3 at prolonged labour may be an explanation of the occurrence of prolonged labour.

The mRNA levels obtained by real time RT-PCR are in accordance with the expression registered by immunohistochemistry, with the highest levels in normal labour. The levels of Connexin 43 mRNA expression showed a tendency to increase at labour. This is in line with earlier investigations where an increase in both the protein level and the number and size of gap junctions at onset of labour have been registered [7,8]. The results in the group with the prolonged labour contradict a previous study by Pierce et al., where it was held that prolonged labour is not related to a reduced expression of the mRNA or the protein level of Connexin 43 [29]. The difference in mRNA expression may be explained by the fact that we used real time PCR, which enables a more exact quantification of mRNA with high fidelity as to the identity of the product. As regards the estimation of the protein expression, it should be stated that immunohistochemistry is not a quantitative method. Nevertheless, our results may indicate that both connexin 43 and syndecan 3 have sufficiently high immunoreactivity in normal labour to be of importance for myometrial contractility. The choice of control group may be open to criticism, since the mean age in that group is higher than the others. However, the only available women are those undergoing hysterectomy. From our analyses of the human cervix, we did not find any differences in the ECM that were related to age, as long as the women still had menstruations (Unpublished data). All women with prolonged labour are given intravenous infusion of oxytocin on a routine basis. The interactions between oxytocin and other mediators involved such as connexin-43 and NO cannot be excluded (14).

The real new important data is that there are both a co-distribution and a co-variation of Connexin 43 and Syndecan 3. Using confocal microscopy, a clear co-localization of Connexin 43 and Syndecan 3 was observed. By focusing on different levels in the tissue, it was shown that the co-localization occurred in the cell membranes. Localization in the cell membranes could possibly play a role in cell-to-cell communication, enhancing the contractile process. Evidence of gap-junctions induced by proteoglycans has been also shown in other systems [17]. This co-variation was also established concerning mRNA-expression. This new finding may imply a combined role for Syndecan 3 and Connexin 43 for the myometrial contractility in normal term labour. In the rat uterus, Syndecan 3 mRNA expression is induced by 17β-estradiol, which further supports the conclusion that syndecan 3 is involved in the myometrial contractility during labour [30]. These results are further supported by new findings in vitro showing ECM-induction of gap junctions in mammary epithelial cells [21].

Mechanical load can also induce gap junction formation and is described in rat myometrium during pregnancy [31]. Mechanical strain in other tissues such as vascular smooth muscle can stimulate the ECM, resulting in an increase of proteoglycan synthesis [32-34]. Therefore, the mechanical load in late pregnancy may be another factor influencing the synthesis of Syndecan 3 at onset of labour.

## Conclusion

The high expression of syndecan 3 and connexin 43 and their co-localization to the smooth muscle bundles during normal labour suggest a functional interaction. Furthermore, the significant reduction in the tissues from women in prolonged labour suggests a role in myometrial contractility. Further studies are needed to ascertain the mechanisms involved.

## Competing interests

The authors declare that they have no competing interests.

## Authors' contributions

AHC selected and recruited patients, collected biopsies, participated in the design of the study, the analysis and interpretation of data and drafted the manuscript. BB participated in the design of the study, the laboratory work, the analysis of data and the drafting of the manuscript. AK participated in the analysis and interpretation of data, did statistical analysis and drafted the manuscript. CD did the confocal microscopy and analysis of the data. GC participated in planning and analyzing real time RT-PCR. AM participated in the design of the study, the analysis and interpretation of the data and critically revised the manuscript. GEO participated in the design of the study, the analysis and interpretation of the data and drafted the manuscript. All authors read and approved the final manuscript.
